# The Control of the Sale of Drink

**Published:** 1919-05-24

**Authors:** 


### The Hospital

The Workers' Newspaper of Administrative Medicine and Institutional Life, Administration, National Insurance and Health.

No. 1720, Vol. LXVI. SATURDAY, MAY 24, 1919. PRICE TWOPENCE

### THE CONTROL OF THE SALE OF DRINK.

At no great distance of time the nation, through its representatives in Parliament, will have to make up its mind what is to be the future policy of the State in regard to the control of the sale of intoxicants. As everyone knows, a degree of State control, State interference, State restriction, upon the ordinary liberties of the citizen to decide for himself how, when, and in what form he should consume alcohol has been maintained during the War to an extent never before practicable in this country. That emergency control has admittedly modified in many respects the habits, in respect of alcoholic drinks; and the industrial efficiency of the people. Whether it shall be extended, abolished, modified, or maintained *in statu quo* during the period of "reconstruction" after the Peace Treaties are signed is a matter of profound importance to everyone of us; and the time is fully ripe for serious consideration of the matter. As a contribution to such consideration the recently published second edition of Mr. Henry Carter's study* of the data now available as to the effects of restriction during the War forms a most valuable aid. The peculiar merit to the economist of this war experience is that for the **first** time partial control of the trade in intoxicants was assumed and directed in the sole interest of national efficiency, as it appeared to the Board of Control. All other factors, such as votes at elections and revenue, were treated as extraneous and disregarded; and therefore conclusions, fairly and logically drawn from data clearly proved, may be acted upon and trusted to a much greater degree than could ever be said before the War, when the idealist, the crank, and the unbalanced social reformer were wont to propound "reforms" which the common sense of the community rejected with emphasis.

It was, indeed, the peculiar irony of facts that reasonable reform of the evils of excessive intemperance in alcohol was chiefly found impossible before the war because of the excessive intemperance in words and ideas of the most earnest advocates of total abstinence. Now, happily, the zeal of the crank "reformer" and the obstruction of the "drink trade" can both be corrected by the experience born of actual practical experiment. It was not to be expected that the Liquor Control Board would make no mistakes; but on the whole it has most decidedly been a blessing to the nation at war, and the lessons of the past few years have only to be wisely acted upon to ensure a much freer, healthier, happier, and more sober British Empire than anyone now alive has ever seen. The present time is a peculiarly favourable one for settling the broad lines of future policy. A Coalition Parliament is surely better suited to deal honestly with such a matter than one elected on party lines. As things are now, members can, and it is to be hoped will, act according to their honest convictions; and provided they will take due trouble to study the whole matter, nothing but good should ensue. When, as in times not so remote, one of the great parties, however anxious its individual members might be to promote real temperance, was habitually supported at elections by the licensed victualling influence; while the other, egged on by the fanatical extremists, introduced measures designed to lead to Prohibition, of which most of its members disapproved, and at the same time its principal club in London was freely stated to consume alcohol in quantities surpassing all other such establishments, there was an unreality and a hypocrisy about the miserable wrangles that perforce ensued that stifled all true progress.

Along what lines, then, should Parliament proceed? Mr. Carter evidently does not believe that Prohibition—"going dry," as they call it in the United States and Canada—is practicable in this country: though his general tone rather suggests that this is the remedy that appeals to him personally. If this correctly represents him, he is to be congratulated on a degree of penetration which not all those who sympathise with him possess. The Hospital has long maintained, as the result of expert commissions appointed early in the century expressly for the purpose, that alcoholic beverages

* "The Control of the Drink Trade." By the Rev. Henry Carter. (Longmans, Green and Co. 4s. 6d. and 2s. 6d. net.)

taken in strict moderation do act beneficially on the human frame both as foods and otherwise; and has, therefore, never adopted the extreme attitude of Prohibition. At the same time, the magnitude of the evils caused by drunkenness in Great Britain has never been minimised in these columns, nor denied. The promotion of real temperance is an object worthy of every man who loves his country. It should not be forgotten that during the War a long stride forward to such temperance has been made; and we are inclined to believe that it has been the no-treating orders and the restrictions upon the hours during which licensed premises may be open that have had a very large share in this happy result. Provided due care is taken to restrict the liberty of the subject as little as is compatible with sobriety, this seems the line upon which immediate progress chiefly depends: more so, at any rate, than upon excessive taxation of beer or the unnecessary dilution of that beverage. It should not be forgotten that even in so-called "bone-dry" States in America it is perfectly simple to evade the law for those who have made up their minds to get intoxicants, a state of affairs which leads to an extensive illicit traffic in very inferior spirits and even in methylated spirits and in *eau de Cologne*—so at least American medical officers testify. Also that the evils of opium, cocaine, heroin and similar drugs are even worse than the evils of drunkenness, and much less easy to uproot; and that quite a proportion of those who can be rendered sober—or less drunken—by legislation break out in this other way and become far more dangerous to themselves and their neighbours than before. Let us, however, not end upon a note of pessimism. We believe that honest legislation, not upon party lines, can in the near future contribute to the diminution of the evils of excessive drinking in this country; and to say that much means that control of the trade, more or less along the lines of the action of the last four years, is necessary in the interests of the nation. Sectarian and party strife, based upon a groundwork of either greed or fanaticism, is to be avoided at all costs; otherwise all attempts at amelioration will fail.

